# Projection of the prevalence of tracheal, bronchus, and lung cancer incidence using cigarette smoking prevalence in Iran from 1990 to 2018: a comparison of latent period-based models with standard forecasting models

**DOI:** 10.1186/s12889-024-19407-8

**Published:** 2024-07-15

**Authors:** Alireza Mirahmadizadeh, Jafar Hassanzadeh, Afrooz Mazidi Moradi, Zahra Gheibi, Alireza Heiran

**Affiliations:** 1 Non-communicable Diseases Research Center, Shiraz University of Medical Sciences, Shiraz, Iran; 2 Research Centre for Health Sciences, Institute of Health, School of Health, Department of Epidemiology, Shiraz University of Medical Sciences, Shiraz, Iran; 3 Student Research Committee, Shiraz University of Medical Sciences, Shiraz, Iran; 4 Department of Epidemiology, Shiraz University of Medical Sciences, Shiraz, Iran

**Keywords:** Lung cancer, Projection, Smoking, Latent period, Time series

## Abstract

**Background:**

Smoking is the major risk factor for tracheal, bronchus, and lung (TBL) cancers. We investigated the feasibility of projecting TBL cancer incidence using smoking incidence rates by incorporating a range of latent periods from the main risk factor exposure to TBL cancer diagnosis.

**Methods:**

In this ecological study, we extracted data on TBL cancer incidence rates in Iran from 1990 to 2018 from the Global Burden of Disease (GBD) database. We also collected data on Iranian cigarette smoking patterns over the past 40 years through a literature review. The weighted average smoking incidence was calculated using a fixed-effects model with Comprehensive Meta-Analysis (CMA) software. Using these data, the five-year TBL cancer incidence in Iran was projected through time series modeling with IT Service Management (ITSM) 2000 software. A second model was developed based on cigarette smoking incidence using linear regression with SPSS (version 22), incorporating different latent periods. The results of these two models were compared to determine the best latent periods.

**Results:**

An increasing trend in TBL cancer incidence was observed from 2019 to 2023 (first model: 10.30 [95% CI: 9.62, 10.99] to 11.42 [95% CI: 10.85, 11.99] per 100,000 people). In the second model, the most accurate prediction was obtained with latent periods of 17 to 20 years, with the best prediction using a 17-year latent period (10.13 to 11.40 per 100,000 people) and the smallest mean difference of 0.08 (0.84%) per 100,000 people using the standard forecasting model (the ARIMA model).

**Conclusion:**

Projecting an increase in TBL cancer incidence rates in the future, an optimal latent period of 17 to 20 years between exposure to cigarette smoke and TBL cancer incidence has implications for macrolevel preventive health policymaking to help reduce the burden of TBL cancer in upcoming years.

**Supplementary Information:**

The online version contains supplementary material available at 10.1186/s12889-024-19407-8.

## Key messages

• The most accurate prediction of five-year TBL cancer incidence in Iran was obtained with latent periods of 17 to 20 years after initial exposure to cigarette smoke.

• A more advanced model can potentially be leveraged to project the epidemiological indices of other non-communicable diseases that have a highly attributable risk factor, adequate information on previous trends, and a range of latent periods.

**Supplementary Information:**

The online version contains supplementary material available at 10.1186/s12889-024-19407-8.

## Introduction

The Global Burden of Disease (GBD) group estimates that approximately 2.3 million new cases of tracheal, bronchus, or lung (TBL) cancer occur annually, with a standardized annual incidence rate of 27.7 per 100,000 people [1], accounting for nearly 1 in 10 cancer cases [2]. In Asia, lung cancer is the most common cancer, accounting for 13.8% of all cancer cases [3]. More than 55% of TBL cancer cases and deaths occur in this region [4]. In Iran, TBL cancer ranks as the fifth and sixth most common cancer among men and women, respectively [5].

TBL cancer has several known risk factors; however, in many communities, the epidemic is primarily driven by a single risk factor: smoking [6]. Smoking is responsible for approximately 80 to 90% of TBL cancer cases in areas where smoking is prevalent. The association between smoking and TBL-related cancer is well documented, with smoking increasing the risk of TBL-related cancer by up to 20 times [7]; therefore, smoking plays a central role as the major TBL-related cancer risk factor, potentially allowing the prediction of TBL-related cancer incidence rates based on smoking prevalence.

Most predictions of TBL cancer rates are grounded in regression models of historical cancer rates. Moreover, there is a temporal lag, often referred to as the latent period, between the onset of smoking and the development of TBL cancer [8], which ranges from 10 to 40 years [9]. Hence, an interesting question is whether a specific latent period range can be established for forecasting TBL cancer cases among cigarette smokers. A study by Davis et al. (2013) [10], revealed that the decreasing trend in lung cancer deaths (1990–2007) in Georgia was associated with a concurrent decrease in smoking incidence (1985–2010) in that region, regardless of the time delay. However, research on whether smoking levels can serve as a proxy for TBL cancer incidence rates in future years is limited. Using a 30-year delay, Seisen et al. (2023) compared the incidence of bladder and lung cancer (1983 and 2013) with the decreasing trend in smoking incidence (1953 to 1983) among people aged 18 and older in the United States. A study showed a stronger correlation between smoking and lung cancer incidence than between smoking and bladder cancer incidence [11]. This study assessed the time delay between smoking and lung cancer mortality, building on previous research findings [12].

The present ecological study sought to determine whether the incidence of TBL cancer in Iran is a function of smoking. We proposed a method contrasting two distinct prediction models for TBL cancer incidence: one using time series modeling of historical TBL cancer rates and the other developed based on the smoking prevalence pattern with varying latent periods using linear regression. We assumed that models with the least mean difference in projected TBL cancer incidence rates represent the most appropriate latent period(s).

## Materials and methods

### Data collection

This ecological study is based on data on TBL cancer incidence and smoking prevalence over the past several years. Data on TBL cancer incidence in Iran from 1990 to 2018 were extracted from the GBD database [8]. To account for the temporal sequence between TBL cancer incidence and smoking status, we collected data on smoking prevalence over the past 40 years (1979 to 2018; 11 years prior) through conducting a systematic database review. We searched for relevant terms, including “smoke/smoking”, “prevalence”, “Iran/Iranian”, and “cigarette”, in both the English (Medline/PubMed, Web of Knowledge, Scopus, and Google Scholar) and Persian (SID, Magiran, Irandoc, Noormags, and Silvica) literatures (Supplementary Fig. 1), as well as the “Atlas of Non-Communicable Diseases Risk-Factors Surveillance”, in the Islamic Republic of Iran (STEPs) in 2005, 2006, 2007, 2009, 2011, and 2016. Studies with a sample size of less than 100, prevalence data that included other substances, or data conducted among specific disease populations (e.g., students) were excluded. After summarizing the results of 283 selected studies out of 1,328 studies, we obtained the most commonly reported index as the target index for our proposed prediction model, which was the percentage of “current smokers”, defined as the percentage of the population aged 15 and older who currently smoke cigarettes [8].

### Calculating smoking prevalence

**Fig. 1:**
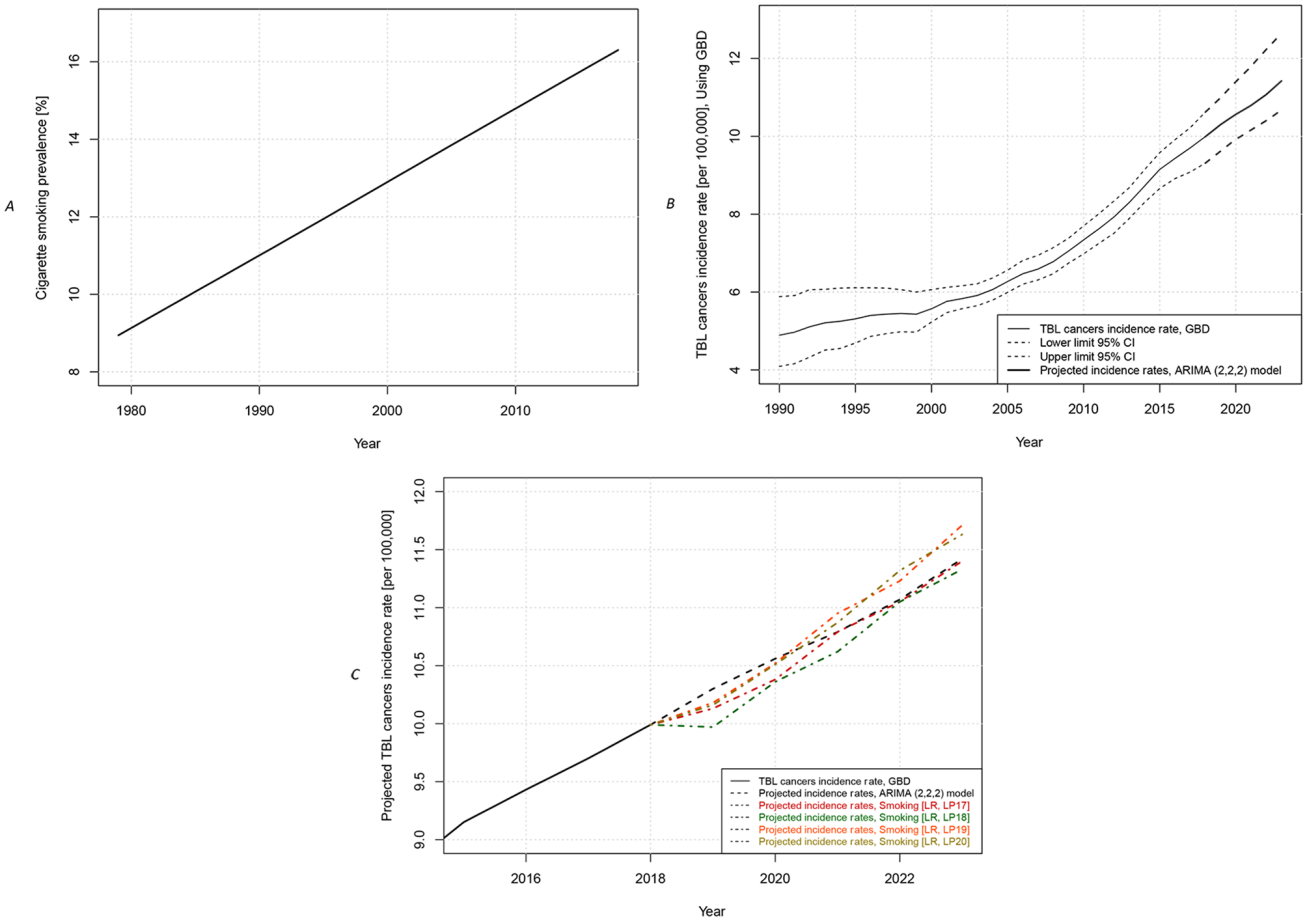
**(A)** Smoothed time trend of current cigarette smoker prevalence during 1979?2018 in Iran; **(B)** Time trend of the TBL cancer incidence rate during 1990?2018 in Iran [GBD data (8)] and the projected rates from 2019 to 2023 using the ARIMA (2,2,2) model; **(C)** Comparison of the projected TBL cancer incidence rates from 2019 to 2023 in Iran, using the ARIMA (2,2,2) model and linear regression (LR) with 17-year, 18-year, 19-year, 20-year latent periods (LP17 to LP20). *Abbreviations* TBL, tracheal, bronchus, and lung; GBD, Global Burden of Disease; CI, confidence interval; LP, latent period; LR, linear regression; ARIMA, Autoregressive Integrated Moving Average

First, if the smoking incidence in a specific calendar year was reported in multiple studies, the weighted average smoking incidence was calculated using the fixed effects model in Comprehensive Meta-Analysis (CMA) software. Second, if only one article reported smoking incidence for a specific calendar year, that single value was used. Third, if data could not be retrieved for a specific calendar year, we calculated the missing value by averaging two values estimated using forward and backward forecasting on the nearest five years with the linear trend command in Microsoft Excel software. By plotting the 40-year trend of smoking incidence after smoothing the data, using the LOWESS smoother in the R programming language (version 4.0.4 for MacOS) (Fig. 1A; Supplementary Table 1), the data were prepared for the prediction phase.

### Statistical analysis

A five-year projection of TBL cancer incidence in Iran was conducted using univariate time series modeling based on TBL cancer incidence rates derived from the GBD data with IT Service Management (ITSM) 2000 software. The second prediction model was developed based on the prepared cigarette smoking prevalence dataset using linear regression with SPSS software (version 22), incorporating various latent periods. The results of these two predictions were subsequently compared. The agreement between smoking incidence and TBL cancer incidence was assessed through sensitivity analysis of latent periods ranging from 10 to 26 years, selected based on the systematic database review (Supplementary Table 2). The highest agreement (least mean difference) in projected values between the two models represented the best latent period for projecting the TBL cancer incidence rate based on the smoking patterns.

## Results

According to the GBD database, the TBL cancer incidence in Iran increased from 4.89 (95% CI: 4.09 to 5.88) in 1990 to 9.99 (95% CI: 9.31 to 10.62) per 100,000 people in 2018. These data were subsequently transformed to achieve the stationarity required for time series analysis (Supplementary Fig. 2). The autocorrelation function (ACF) and partial autocorrelation function (PACF) diagrams were used to estimate the parameters of the first projection model, i.e., *p* and *q*, which yielded *p* = 3 and *q* = 8, respectively (Supplementary Fig. 3). After testing the different time series models, the ARIMA (2,2,2) model was found to be the best projection model, with an Akaike information criterion (AIC) coefficient of -73.74. The final model equation was *X*_*t*_ = 0.3765*X*_*(t—k)*_ — 0.9768 *X*_*(t—2k)*_ + *Z*_*(t)*_ — 0.5763 *Z*_*(t—k)*_ + 0.9998 *Z*_*(t—2k)*_, where X_t_ represents the TBL cancer incidence rate at year (time point) *t*, *X*_t−k_ represents the TBL cancer incidence rate *k* years before year *t*, Z_t_ indicates the random error value at year *t*, and Z_t−k_ indicates the random error value at *k* years before year *t*.

The fitness of the model was further evaluated, showing no correlation between the model’s residuals. The residuals were also nearly normally distributed, as observed in the Q‒Q plot and histogram (Supplementary Fig. 4). Based on the ARIMA (2,2,2) time series analysis from 1990 to 2018, the TBL cancer incidence was projected as follows: 10.30 [95% CI: 9.62, 10.99], 10.56 [95% CI: 9.92, 11.40], 10.79 [95% CI: 10.16, 11.79], 11.07 [95% CI: 10.40, 12.23], and 11.42 [95% CI: 10.67, 12.64] per 100,000 Iranian people from 2019 to 2023, indicating an increasing trend (Fig. 1B).

Table 1 shows the projected TBL cancer incidence rates from 2019 to 2023 using the cigarette smoking prevalence dataset, considering latent periods of 10 to 26 years, as generated by the second model. The highest agreement in projected values between the two models was obtained at latent periods of 17 to 20 years, with mean differences of 0.08 (0.84%), 0.16 (1.48%), 0.15 (1.39%), and 0.15 (1.39%) per 100,000 people, respectively, representing the best latent periods for projecting the TBL cancer incidence rate based on smoking patterns in Iran (Fig. 1C). Notably, the latent period of 17-year provided the most accurate forecast, showing an increasing trend (10.13 in 2019 to 11.40 per 100,000 people in 2023).

**Table 1:** Projected TBL cancer incidence rates (per 100,000 people) in 2019 to 2023 using a linear regression model and sensitivity analysis on cigarette smoking prevalence at different latent periods in comparison with a standard forecasting model (ARIMA (2,2,2) model) based on GBD data

Year	Predicted by ARIMA (2,2,2) model	Projected TBL cancer rates using linear model on cigarette smoking prevalence at different (10–26 years) latent periods																
		10 y	11 y	12 y	13 y	14 y	15 y	16 y	17 y	18 y	19 y	20 y	21 y	22 y	23 y	24 y	25 y	26 y
2019	10.30 [9.62, 10.99]	9.44	9.46	9.70	9.63	9.76	9.83	9.79	10.13	9.97	10.18	10.16	10.32	10.45	10.28	10.53	10.32	10.55
2020	10.56 [9.92, 11.40]	9.68	9.70	9.97	9.89	10.04	10.05	10.16	10.38	10.36	10.52	10.51	10.70	10.73	10.76	10.85	10.82	10.96
2021	10.79 [10.16, 11.79]	9.88	9.95	10.24	10.17	10.32	10.34	10.40	10.79	10.62	10.95	10.87	11.07	11.14	11.05	11.38	11.15	11.52
2022	11.07 [10.40, 12.23]	10.19	10.16	10.51	10.44	10.62	10.64	10.71	11.05	11.05	11.23	11.32	11.46	11.54	11.48	11.69	11.70	11.88
2023	11.42 [10.67, 12.64]	10.39	10.47	10.73	10.73	10.92	10.96	11.03	11.40	11.33	11.71	11.63	11.95	11.95	11.91	12.17	12.04	12.47
**Mean difference between ARIMA (2**,**2**,**2) model and linear regression projected values**		0.91 (8.4%)	0.88 (8.13%)	0.60 (5.54%)	0.66 (6.1%)	0.50 (4.62%)	0.46 (4.25%)	0.41 (3.79%)	0.08 (0.74%)	0.16 (1.48%)	0.15 (1.39%)	0.15 (1.39%)	0.27 (2.49%)	0.33 (3.05%)	0.27 (2.49%)	0.49 (4.53%)	0.38 (3.51%)	0.66 (6.1%)

*Abbreviations * TBL, tracheal, bronchus, and lung; GBD, Global Burden of Disease; ARIMA, Autoregressive Integrated Moving Average; y, year

## Discussion

With the lack of lung cancer registries in many regions, several studies have been conducted to project lung cancer rates through proxy estimators. These studies generally project lung cancer mortality or incidence (less than the former), with a cigarette smoking index as the proxy and with the lag (latent) period(s) using various models, such as generalized linear models (GLMs), age-period-cohort (APC) models, polynomial distributed lag (PDL) models, distributed lag nonlinear models (DLNMs), often followed by validation. A summary of these studies and their methods for projecting lung cancer mortality or incidence are yielded in Supplementary Table 3. The key idea of our method was to investigate whether the incidence of TBL cancer, a chronic disease, can be projected based on the pattern of its primary risk factor, cigarette smoking, incorporating the best latent period.

Based on GBD data, the TBL cancer incidence in Iran has been increasing, which is consistent with the findings of other studies conducted in Iran. For example, Khanali et al. (2021) reported an average annual increase in TBL cancer incidence rates of 6.8% for men and 7.7% for women during 2000–2016 based on the National Cancer Registration Program (NCRP) [13]. In addition, Roshandel et al. (2021) showed that the age-standardized incidence rate of TBL cancer increased from 7.3 per 100,000 people in 2008 to 8.2 per 100,000 people in 2016 [14].

We also found that the cigarette smoking prevalence was increasing in Iran, in contrast to the decreasing trend observed in several developed countries, such as the United States and Canada [9]. Through linear regression modeling of cigarette smoking incidence and sensitivity analysis of latent periods ranging from 10 to 26 years, an increasing trend in the TBL cancer incidence rate was identified. Generally, when considering the latent period of 17 to 20 years, our method was able to predict Iran’s TBL cancer incidence rate during the 2019–2023 using the cigarette smoking prevalence with remarkable accuracy. This method contrasts with standard forecasting models, which have a mean difference of approximately 0.08 (0.84%) per 100,000 people. Interestingly, our identified latent period range was relatively consistent with or near previous research from different countries, such as 18 years in Lipfert et al. (2019) [15], 21 years in the study of Peace (1985) [16], and 20 years in Alberg et al. (2013) [17], and was different from the 25–30 years reported by Shibuya et al. (2005) [18]. These variations might be attributed to difference in projection models, smoking and tobacco consumption definitions, and other factors [18]. Overall, we were able to confirm the main idea of the study, at least with our limited and specific data; that is, using smoking prevalence rates and mathematical models, we could obtain accurate estimates of future TBL incidence rates.

The primary risk factor for lung cancer is smoking, which has a high prevalence in societies [6]. Therefore, communities that have successfully decreased tobacco use through the implementation of appropriate preventive and control programs have experienced decreases in TBL cancer incidence and mortality, with varying delays [18]. For example, in the United States, tobacco use was effectively controlled through strategies such as smoking restrictions in public places, raising cigarette taxes, decreasing access to cigarettes, and public awareness campaigns on the dangers of smoking [19], which prevented approximately 146,000 male lung cancer deaths between 1991 and 2003 [20]. Conversely, implementing lung cancer screening programs, particularly low-dose computed tomography (LDCT), among high-risk groups, as determined by the justified latent period (adults aged 50 to 80 years who have a 20-pack-year smoking history and either currently smoke or have quit within the past 15 years), helps reduce lung cancer deaths in high-income countries [21].

Our study had at least five major limitations: (1) lack of a national registry for continuous monitoring of smoking status in Iran; (2) inadequate data on smoking prevalence in most years of the studied period in the region; (3) the presence of heterogeneity in selected studies according to the systematic database review in terms of sample size, age group, and target population; (4) an inability to project TBL cancer mortality rates using the proposed method due to limited literature; and (5) the inherent limitations associated with GBD data, which was one of our references during the data collection, specifically the curse of the availability of primary data [22].

### Conclusion

A latent period of 17 to 20 years between exposure to cigarette smoke and the incidence of TBL cancer might be a valuable piece of knowledge for helping macrolevel health decision makers implement preventive and control programs for tobacco use and guide TBL cancer screening, specifically LDCT, programs for high-risk groups (according to the justified latent period) to reduce the burden of TBL cancer in Iran in the upcoming years. Additionally, a more advanced and robust model can potentially be leveraged to project the epidemiological indices of other noncommunicable diseases that have a highly attributable risk factor, available historical trend information, and a range of latent periods.

### Electronic supplementary material

Below is the link to the electronic supplementary material.

Supplementary Material 1

## Data Availability

The 40-year smoking prevalence in the Iranian dataset generated during the current study is available from the corresponding author upon reasonable request. In addition, the dataset analyzing the incidence of TBL cancer in Iran during the current study is available in the International Health Organization (IHME) Global Burden of Disease (GBD) repository, [https://www.healthdata.org/gbd/data-visualizations].
