# Supplementary material for: Projection of the prevalence of tracheal, bronchus, and lung cancer incidence using cigarette smoking prevalence in Iran from 1990 to 2018: a comparison of latent period-based models with standard forecasting models
Source: BMC Public Health. 2024 Jul 15;24:1896. doi: 10.1186/s12889-024-19407-8 (PMC11251385; doi:10.1186/s12889-024-19407-8)
Supplement: Supplementary file 1 — Supplementary Material 1 [file 12889_2024_19407_MOESM1_ESM.docx]

**Identification of studies via databases and registers**

Records removed *before screening*:

Duplicate records removed (n = 254)

Records marked as ineligible by automation tools (n = NA)

Records removed for other reasons (n = 0)

Records identified from:

Databases (n = 1328)

Registers (n = 0)

**Identification**

Records screened

(n = 1074)

Records excluded

(n = 545)

Reports sought for retrieval

(n = 529)

Reports not retrieved

(n = 0)

**Screening**

Reports assessed for eligibility

(n = 529)

Reports excluded:

Irrelevant (n = 238)

Conference report (n = 6)

Poster report (n = 2)

etc.

Studies included in review

(n = 283)

Reports of included studies

(n = 283)

**Included**

**Supplementary Figure 1.** PRISMA 2020 flow diagram for the systematic review and meta-analysis on the 40-year smoking prevalence in Iran

**Supplementary Figure 2.** Time series of TBL cancers incidence rate in Iran from 1990 to 2018 after data transformation for stationarity


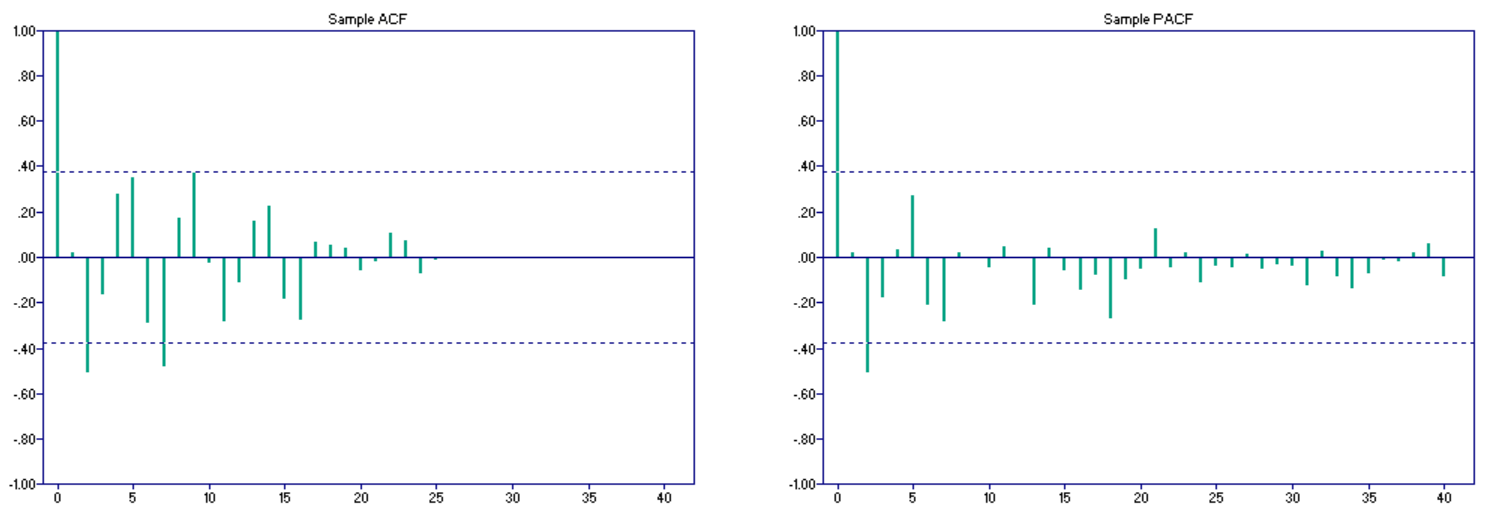


**Supplementary Figure 3.** PAC and PACF diagrams derived from the stationery data

| *A* |  |
| --- | --- |
| *B* |  |
| **Tests of randomness on residuals**  *Ljung - Box statistic:* Chi-Square (20) = 20.985, *P* = 0.39802  *McLeod - Li statistic:* Chi-Square (24) = 18.466, *P* = 0.77983  *# Turning points:* 18.000 ~ AN(16.667, SD = 2.1161), *P* = 0.52863  *# Diff sign points:* 12.000 ~ AN(13.000, SD = 1.5275), *P* = 0.51269  *Rank test statistic:* 0.18200E+03 ~ AN(.17550E+03, SD = 23.984), *P* = 0.78638  *Jarque-Bera test statistic (for normality):* Chi-Square (2) = 0.042679, *P* = 0.97889  *Order of Min AICC YW Model for Residuals:* 0 | |
| **Supplementary Figure 4.** Assessing the fitness of time series model using the tests evaluating the normality of the residuals’ distribution; *(A)* histogram, *(B) Q-Q* plot | |

| **Supplementary Table 1.** Smoking prevalence (Percentage of current smokers) during 1979-2018 in Iran | | | |
| --- | --- | --- | --- |
| Year | Number of articles | Arithmetic average | Weighted average |
| 1979 | 0 | NaN | 8.9 ^1^ |
| 1980 | 0 | NaN | 9.1 ^1^ |
| 1981 | 0 | NaN | 9.3 ^1^ |
| 1982 | 0 | NaN | 9.5 ^1^ |
| 1983 | 0 | NaN | 9.7 ^1^ |
| 1984 | 0 | NaN | 9.7 ^1^ |
| 1985 | 0 | NaN | 9.9 ^1^ |
| 1986 | 0 | NaN | 10.3 ^1^ |
| 1987 | 0 | NaN | 10.5 ^1^ |
| 1988 | 0 | NaN | 9.9 ^1^ |
| 1989 | 0 | NaN | 10.2 ^1^ |
| 1990 | 1 | 11.9 | 11.17 ^2^ |
| 1991 | 1 | 14.6 | 12.2 ^2^ |
| 1992 | 1 | 16.54 | 13.01 ^2^ |
| 1993 | 2 | 18.21 | 11.6 ^2^ |
| 1994 | 1 | 18.4 | 13.6 ^2^ |
| 1995 | 0 | NaN | 11.5 ^1^ |
| 1996 | 2 | 11.44 | 11.5 ^2^ |
| 1997 | 0 | NaN | 11.9 ^1^ |
| 1998 | 2 | 9.02 | 9.5 ^2^ |
| 1999 | 7 | 12.8 | 12.1 ^3^ |
| 2000 | 5 | 14.24 | 14.6 ^3^ |
| 2001 | 10 | 13.77 | 14.2 ^3^ |
| 2002 | 7 | 14.15 | 14.5 ^3^ |
| 2003 | 6 | 13.21 | 13.4 ^3^ |
| 2004 | 10 | 12.98 | 13.1 ^3^ |
| 2005 | 9 | 17.39 | 17.4 ^3^ |
| 2006 | 9 | 11.43 | 11.7 ^3^ |
| 2007 | 11 | 11.93 | 12 ^3^ |
| 2008 | 10 | 11.77 | 11.9 ^3^ |
| 2009 | 12 | 12.13 | 12.3 ^3^ |
| 2010 | 10 | 15.51 | 15.9 ^3^ |
| 2011 | 8 | 15.07 | 15.5 ^3^ |
| 2012 | 14 | 14.93 | 16.7 ^3^ |
| 2013 | 18 | 15.65 | 17.1 ^3^ |
| 2014 | 27 | 13.67 | 14 ^3^ |
| 2015 | 14 | 16.8 | 11 ^3^ |
| 2016 | 19 | 15.98 | 16.3 ^3^ |
| 2017 | 14 | 13.27 | 13.6 ^3^ |
| 2018 | 12 | 15.8 | 17.2 ^3^ |
| ^1^ Estimated using forward and backward forecasting on the nearest 5 years  ^2^ Prevalence was reported in only one article  ^3^ Calculated using the fixed-effect model | | | |

| **Supplementary Table 2.** Data summary of the latent periods from the smoking and TBL cancers incidence through the systematic database analysis | | | |
| --- | --- | --- | --- |
| **#** | **First author, publication year, country** | **Latent period, *y*** | **Index** |
| 1 | Saba, 2015, Iran | 10 | Cigarette smoking prevalence |
| 2 | Qi, 2021, China | 20-30 | Smoking impact ratio (SIR) |
| 3 | Kong, 2021, NA | 10-20 | Cigarette smoking prevalence |
| 4 | Winkler, 2015, South Africa | 10-20 | Cigarette smoking prevalence |
| 5 | Luo, 2019, Australia | 20-30 [optimal: 25] | Smoking prevalence and cigarette tar exposure per capita |
| 6 | Lipfert, 2019, NA | 18 | Annual cigarette sales |
| 7 | Peace, 1985, UK | 21 | Cigarette sales |
| 8 | Alberg, 2013, multiple | 20 | Currently smokers prevalence |
| 9 | Islami, 2015, multiple | 20-30 | Cigarette smoking prevalence |
| 10 | Weiss, 1976, US | 10-24 | Cigarette smoking prevalence |
| 11 | Weiss, 1997, US | ~30 | Cigarette smoking prevalence |
| 12 | Thun, 2012, multiple | [M] 20-30 | Increase and decrease of cigarette smoking prevalence |
| 13 | Yamaguchi, 2021, Japan | 15 | Cumulative cigarette consumption |
| 14 | Villeneuve, 1994, Canada | NA | Currently smokers and Never smoked |
| 15 | Smith, 2021, multiple | [Min effect] 16  [Max effect] 6 | Cigarette smoking prevalence |
| 16 | Shibuya, 2005, multiple | 25-30 [optimal: 25] | Average tar content and adult cigarette consumption per capita |
| 17 | Schöllnberger, 2006, Italy | 5 | Smoking rates |
| 18 | Pierce, 2010, US | 5-28 [optimal: 21] | Rate of decline in cigarette consumption |
| 19 | Nadler, 2014, US | 13.6 | NA |
| 20 | Li, 2021, China | 10 | Annual per capita tobacco consumption |
| 21 | Leffondré, 2006, Canada | 20 | Rate of decline in currently cigarette smokers |
| 22 | Ito, 2019, multiple | [Filtered cigarettes] 15-25  [Non-filtered] 20-30 | Smoking rates |
| 23 | Dreassi, 2005, Italy | 5, 10, 15 | Socioeconomic factors |
| 24 | Bruder, 2018, Switzerland | 10 | Cigarette smoking status |
| 25 | Brown, 1988, US | 24 | Cigarette smoking prevalence |
| **References, in order:**   1. Saba V. Estimation of Age Standardized Ratio of Lung Cancer in Iran in 2014 and 2030. Paramedical Sciences and Military Health. 2015;10(1):17-23. 2. Qi F, Xu Z, Zhang H, Wang R, Wang Y, Jia X, et al. Predicting the mortality of smoking attributable to cancer in Qingdao, China: A time-series analysis. PLOS ONE. 2021;16(1):e0245769. 3. Kong KA, Jung-Choi KH, Lim D, Lee HA, Lee WK, Baik SJ, et al. Comparison of Prevalence- and Smoking Impact Ratio-Based Methods of Estimating Smoking-Attributable Fractions of Deaths. Journal of epidemiology. 2016;26(3):145-54. 4. Winkler V, Mangolo NJ, Becher H. Lung cancer in South Africa: a forecast to 2025 based on smoking prevalence data. BMJ Open. 2015;5(3):e006993. 5. Luo Q, Yu XQ, Wade S, Caruana M, Pesola F, Canfell K, et al. Lung cancer mortality in Australia: Projected outcomes to 2040. Lung Cancer. 2018;125:68-76. 6. Lipfert FW, Wyzga RE. Longitudinal relationships between lung cancer mortality rates, smoking, and ambient air quality: a comprehensive review and analysis. Critical reviews in toxicology. 2019;49(9):790-818. 7. Peace LR. A Time Correlation Between Cigarette Smoking and Lung Cancer. Journal of the Royal Statistical Society Series D (The Statistician). 1985;34(4):371-81. 8. Alberg AJ, Brock MV, Ford JG, Samet JM, Spivack SD. Epidemiology of lung cancer: Diagnosis and management of lung cancer, 3rd ed: American College of Chest Physicians evidence-based clinical practice guidelines. Chest. 2013;143(5 Suppl):e1S-e29S. 9. Islami F, Torre LA, Jemal A. Global trends of lung cancer mortality and smoking prevalence. Translational lung cancer research. 2015;4(4):327-38. 10. Weiss W. Chloromethyl ethers, cigarettes, cough and cancer. Journal of occupational medicine : official publication of the Industrial Medical Association. 1976;18(3):194-9. 11. Weiss W. Cigarette smoking and lung cancer trends. A light at the end of the tunnel? Chest. 1997;111(5):1414-6. 12. Thun M, Peto R, Boreham J, Lopez AD. Stages of the cigarette epidemic on entering its second century. Tobacco Control. 2012;21(2):96-101. 13. Yamaguchi N, Mochizuki-Kobayashi Y, Utsunomiya O. Quantitative relationship between cumulative cigarette consumption and lung cancer mortality in Japan. International journal of epidemiology. 2000;29(6):963-8. 14. Villeneuve PJ, Mao Y. Lifetime probability of developing lung cancer, by smoking status, Canada. Canadian journal of public health = Revue canadienne de sante publique. 1994;85(6):385-8. 15. Smith DR, Behzadnia A, Imawana RA, Solim MN, Goodson ML. Exposure–lag response of smoking prevalence on lung cancer incidence using a distributed lag non-linear model. Scientific Reports. 2021;11(1):14478. 16. Shibuya K, Inoue M, Lopez AD. Statistical modeling and projections of lung cancer mortality in 4 industrialized countries. International journal of cancer. 2005;117(3):476-85. 17. Schöllnberger H, Manuguerra M, Bijwaard H, Boshuizen H, Altenburg HP, Rispens SM, et al. Analysis of epidemiological cohort data on smoking effects and lung cancer with a multi-stage cancer model. Carcinogenesis. 2006;27(7):1432-44. 18. Pierce JP, Messer K, White MM, Kealey S, Cowling DW. Forty Years of Faster Decline in Cigarette Smoking in California Explains Current Lower Lung Cancer Rates. Cancer Epidemiology Biomarkers &amp; Prevention. 2010;19(11):2801-10. 19. Nadler DL, Zurbenko IG. Estimating Cancer Latency Times Using a Weibull Model. Advances in Epidemiology. 2014;2014:746769. 20. Li L, Lu J, Dai X, Ma L, Wang C, Feng L. The lag effect of 24-year tobacco consumption on lung cancer mortality in Henan Province, China, 1992 to 2016. Environmental Science and Pollution Research. 2021. 21. Leffondré K, Abrahamowicz M, Xiao Y, Siemiatycki J. Modelling smoking history using a comprehensive smoking index: application to lung cancer. Statistics in Medicine. 2006;25(24):4132-46. 22. Ito H, Matsuo K, Tanaka H, Koestler DC, Ombao H, Fulton J, et al. Nonfilter and filter cigarette consumption and the incidence of lung cancer by histological type in Japan and the United States: Analysis of 30-year data from population-based cancer registries. International journal of cancer. 2011;128(8):1918-28. 23. Dreassi E, Biggeri A, Catelan D. Space-time models with time-dependent covariates for the analysis of the temporal lag between socioeconomic factors and lung cancer mortality. Statistics in Medicine. 2005;24(12):1919-32. 24. Bruder C, Bulliard JL, Germann S, Konzelmann I, Bochud M, Leyvraz M, et al. Estimating lifetime and 10-year risk of lung cancer. Preventive medicine reports. 2018;11:125-30. 25. Brown CC, Kessler LG. Projections of Lung Cancer Mortality in the United States: 1985–2025. JNCI: Journal of the National Cancer Institute. 1988;80(1):43-51. | | | |

| **Supplementary Table 3.** Summary of the methods previopusly used to project lung cancer mortality or incidence rates using a cigarette smoking index |
| --- |
| **Summary of method** |
| - Obtaining cancers causually related to smoking - Projecting mortality rate using the previous trend including lagged cigarette tar exposure ^1^ or cigarette smoking exposure ^2^ as a covariate - Using BIC for selecting the most appropriate model - Multiplying mortality rates by the smoking-attributable fraction (SAF ^3^) to calculate the proportion of deaths that are directly attributable to smoking **[1]** |
| - Estimating age-specific lung cancer mortality in non-smokers from published studies ^4^ - Estimating Smoking prevalence data by dose-specific smoking categories (smoking patterns) with their corresponding estimated relative risk from the literature - Obtaining population data - Combining these three figures **[2, 3]** - Extending these smoking dose categories to obtain more accurate estimation **[4, 5]** |
| - Identifying tobacco-related cancers (based on the GBD for example) - Obtaining the cancer death data from a reporting system - Calculating the smoking impact ratio (SIR) - Calculating cancers' smoking attributable mortality (SAM) rate as the function of standard population attributable fraction (PAF) (SIR was used to calculate PAF) - Projecting mortality rate using the time series analysis  ^5^ - Using BIC and the coefficient of determination (R^2^) for selecting the most appropriate model **[6]** |
| - Obtaining and matching age- and sex-specific lung cancer incidence rates and smoking prevalence estimates - Modeling the temporal dependency between changes in smoking prevalence and lung cancer incidence, using a distributed lag non-linear model (DLNM) - Presenting the effects as the incidence rate ratio (IRR) and cumulative incidence rate ratio (IRR_cum_) **[7]** |
| - Obtaining the tobacco consumption rate, as well as the average tar content, and the lung cancer mortality rate - Using the Age-Period-Cohort (APC) or polynomial distributed lags (PDLs) models, optimaized by a specific lag period **[8-10]** |
| **Note:**  ^1^ Generalised Linear Model (GLM) models  ^2^ APC models  ^3^ SAF can be calculated through SIR-based method or prevalence-based approaches with or withought considering exposure-event lag **[11]**  ^4^ Linear Regression (LR) models  ^5^ Autoregressive Integrated Moving Average (ARIMA) models  **References:**   1. Luo Q, Yu XQ, Wade S, Caruana M, Pesola F, Canfell K, et al. Lung cancer mortality in Australia: Projected outcomes to 2040. Lung Cancer. 2018;125:68-76. 2. Ng N, Winkler V, Van Minh H, Tesfaye F, Wall S, Becher H. Predicting lung cancer death in Africa and Asia: differences with WHO estimates. Cancer Causes Control. 2009;20(5):721-30. 3. Bruder C, Bulliard JL, Germann S, Konzelmann I, Bochud M, Leyvraz M, et al. Estimating lifetime and 10-year risk of lung cancer. Preventive medicine reports. 2018;11:125-30. 4. Winkler V, Ng N, Tesfaye F, Becher H. Predicting lung cancer deaths from smoking prevalence data. Lung Cancer. 2011;74(2):170-7. 5. Winkler V, Mangolo NJ, Becher H. Lung cancer in South Africa: a forecast to 2025 based on smoking prevalence data. BMJ Open. 2015;5(3):e006993. 6. Qi F, Xu Z, Zhang H, Wang R, Wang Y, Jia X, et al. Predicting the mortality of smoking attributable to cancer in Qingdao, China: A time-series analysis. PLOS ONE. 2021;16(1):e0245769. 7. Smith DR, Behzadnia A, Imawana RA, Solim MN, Goodson ML. Exposure–lag response of smoking prevalence on lung cancer incidence using a distributed lag non-linear model. Scientific Reports. 2021;11(1):14478. 8. Shibuya K, Inoue M, Lopez AD. Statistical modeling and projections of lung cancer mortality in 4 industrialized countries. International journal of cancer. 2005;117(3):476-85. 9. Li L, Lu J, Dai X, Ma L, Wang C, Feng L. The lag effect of 24-year tobacco consumption on lung cancer mortality in Henan Province, China, 1992 to 2016. Environmental Science and Pollution Research. 2021. 10. Brown CC, Kessler LG. Projections of Lung Cancer Mortality in the United States: 1985–2025. JNCI: Journal of the National Cancer Institute. 1988;80(1):43-51. 11. Kong KA, Jung-Choi KH, Lim D, Lee HA, Lee WK, Baik SJ, Park SH, Park H. Comparison of Prevalence- and Smoking Impact Ratio-Based Methods of Estimating Smoking-Attributable Fractions of Deaths. J Epidemiol. 2016;26(3):145-54. |
